# MEDICAL MORBIDITY RISK IN A SAMPLE OF OLDER WOMEN WITH BINGE EATING

**DOI:** 10.1093/geroni/igac059.1744

**Published:** 2022-12-20

**Authors:** Savannah Hooper, Victoria Marshall, Preston Bridges, Sara Espinoza, Pamela Keel, Andrea LaCroix, Nicolas Musi, Lisa Kilpela

**Affiliations:** UT Health Science Center San Antonio, San Antonio, Texas, United States; UT Health Science Center San Antonio, San Antonio, Texas, United States; The University of Texas Health Science Center San Antonio, San Antonio, Texas, United States; Sam and Ann Barshop Institute, UT Health San Antonio, San Antonio, Texas, United States; Florida State University, Tallahassee, Florida, United States; University of California, San Diego, La Jolla, California, United States; UT Health Science Center San Antonio, San Antonio, Texas, United States; UT Health Science Center San Antonio, San Antonio, Texas, United States

## Abstract

Binge eating (BE; consuming an abnormally large amount of food in one sitting while feeling out of control) is the most common form of disordered eating among older women, and aging may confer greater risk for medical morbidities. While the clinical phenotype of older women with BE is unknown, understanding health impacts of BE on the aging process is important. The objective of this study was to describe physiological characteristics of older women (aged 60+) with clinical levels of BE (i.e., ≥weekly episodes). Participants (N = 21; M age = 66.0±4.59) underwent a DEXA, indirect calorimetry, anthropometric measurements, and fasting blood labs. 76% of participants reported a BE age of onset in midlife or later (age 42+); the sample mean BMI was 35.08±8.64, with an average waist-to-hip ratio of 0.90±.047. DEXA scans indicated 90.48% were classified as overweight or obese. Using age- and gender-matched norms, mean fat percentile was 84.75±17.43; mean total body fat percentage was 47.0±4.5. Mean Resting Energy Expenditure (kcal/lean mass) was 34.2±3.71; average respiratory quotient was 0.81±0.06. HbA1c values indicated that 61.90% had pre-diabetes or diabetes; 47.61% had high fasting blood glucose and 76% had high LDL cholesterol. Mean bone mineral density (BMD) fell within a healthy range (1.11 g/cm2±.14); 33.33% had a BMD z-score of less than zero. Cardiovascular comorbidities were rare in this sample (9.5% had standard hypertension). Overall, metabolic disturbances were prevalent, while a minority had cardiovascular or skeletal morbidities. Findings suggest that BE may interfere with healthy aging predominantly through metabolic morbidities.

